# Can focused echocardiographic evaluation in life support (FEEL) be used to predict resuscitation outcome or termination of resuscitation (TOR)? A prospective trial

**DOI:** 10.1186/2036-7902-7-S1-A24

**Published:** 2015-03-09

**Authors:** JY Suh, YS Jo, JH Choi, HM Kim

**Affiliations:** Emergency medicine, Soonchunhyung University Bucheon Hospital, Bucheon, South Korea

**Keywords:** Cardiac Arrest, Receiver Operating Curve, Cerebral Performance Category, Cardiac Arrest Patient, Hospital Arrival

## Background

There are many studies about predictive factor of return of spontaneous circulation (ROSC) during the cardio-pulmonary resuscitation (CPR), but no definite guideline or predictive factor for termination of CPR.

## Objective

The purpose of this study is to figure out the relationship between ROSC and cardiac activity findings by echocardiography(echo) in cardiac arrest patients, and investigate the cardiac standstill period can be used as indicator for termination resuscitation.

## Patients and methods

We performed a prospective, observational study of non-consecutive, non-trauma, adult, out of hospital cardiac arrest (OHCA) patients. This study conducted in emergency department of single tertiary university hospital. Echo performed every 2 minute simultaneously with pulse check within 10 second throughout the arrest, which was managed by the usual advanced cardiac life-support treatment guidelines. We obtained sub-xiphoid or parasternal long axis view of echo. We defined echocardiographic evidence of cardiac kinetic activity as any detected motion of the myocardium, ranging from visible ventricular fibrillation to coordinated ventricular contractions.

## Results

**Figure 1:**
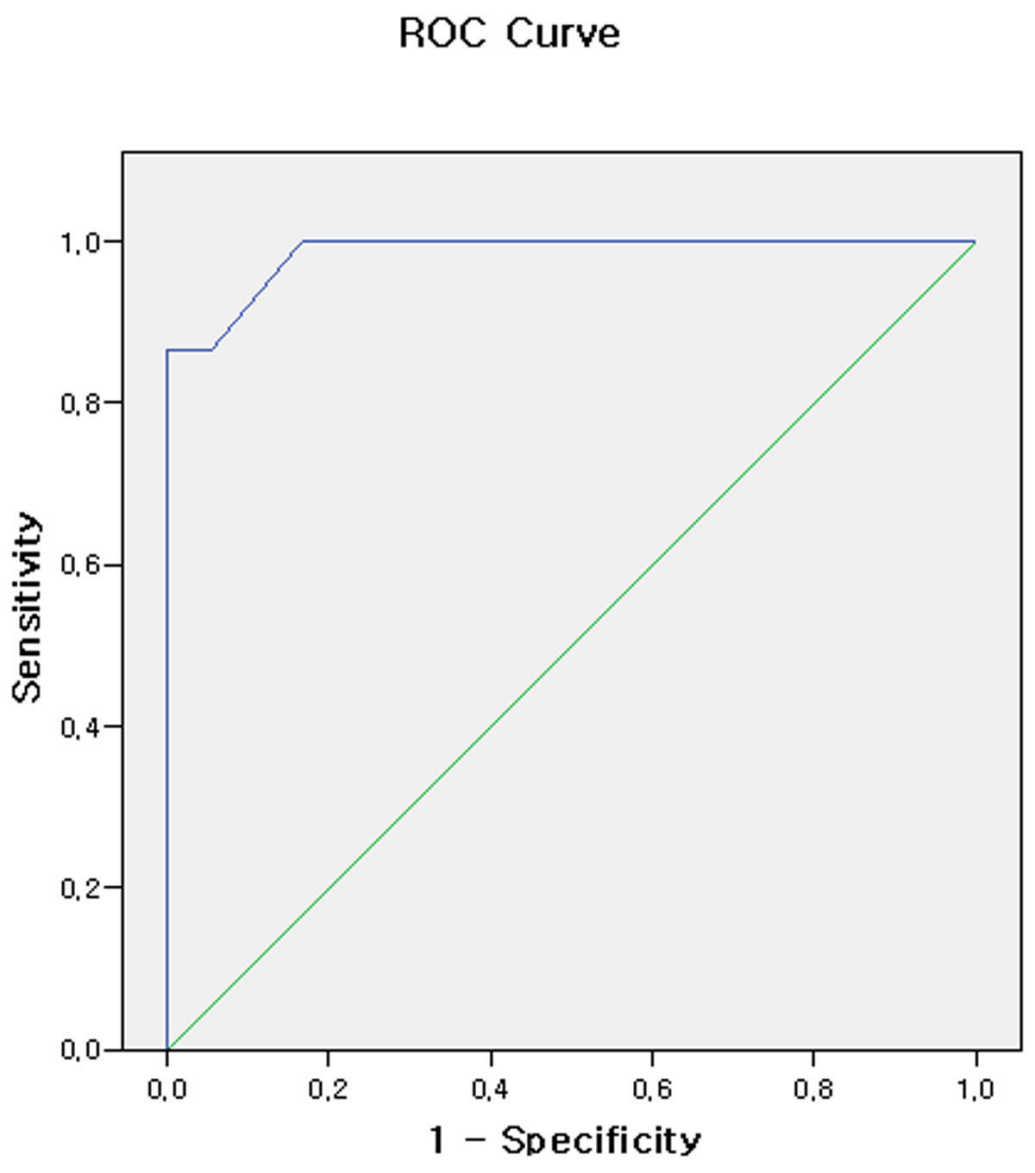
Figure 1

During 10 months, 33 patients were enrolled in the study. Witness arrest patients were 22 (66.7%), 12 (36.3%) patients received bystander CPR. Interval from EMS call to EMS scene arrival is 8±3 minutes, Interval from EMS call to EMS hospital arrival is 23±6 minutes. 17 (51.5%) patients attained ROSC, and 1 (3%) patient discharged in cerebral performance category 1. Repetitive cardiac standstill periods in ROSC group and no ROSC group were 2.9±2.4 minutes versus 18.9±8.4 minutes, respectively (p<0.001). Cardiac standstill period over 8 minutes predicted eventual no ROSC with a sensitivity of 86.7% and a specificity of 100%. A receiver operating curve was generated to determine the accuracy of echocardiographic cardiac standstill period for predicting no ROSC. The Area under the ROC curve was 0.985 (p<0.01). Figure 1.

## Conclusion

If over 8 minutes repetitive cardiac standstill was identified by echo during the CPR, termination of resuscitation could be considered.

